# MENTAL HEALTH RISKS SIGNIFIED BY THE CO-EXPERIENCE OF ELDER MISTREATMENT AND SOCIAL ISOLATION

**DOI:** 10.1093/geroni/igad104.3467

**Published:** 2023-12-21

**Authors:** Juyoung Park, Yuri Jang, Maria Aranda, Shinyi Wu, Hans Oh, Kathleen Wilber

**Affiliations:** University of Southern California, Los Angeles, California, United States; University of Southern California, Los Angeles, California, United States; University of Southern California, Los Angeles, California, United States; University of Southern California, Los Angeles, California, United States; University of Southern California, Los Angeles, California, United States; University of Southern California, Los Angeles, California, United States

## Abstract

Both elder mistreatment (EM) and social isolation are widely known to pose a significant risk to mental health of older individuals. Despite the general agreement that EM should be addressed in consideration of social contexts, there has been limited attention to the potential harm of the co-experience of EM and social isolation. The issue is of particular concern in older Asian immigrants. Using a sample of older Korean Americans (N = 2,150, Mean age = 73.4), we examined the direct and interactive effect of EM and social isolation on mental distress. In a measure including psychological, physical, and financial mistreatment, 32% reported a single EM exposure and 12% multiple EM exposure in the past year. The rate of social isolation, measured with Lubben Social Network Scale-6, was 24%. About 30% were in the category of mental distress measured with Kessler 6. The direct effects of a single EM or multiple EM exposure in reference to no EM and social isolation were significant. In addition, the interaction between multiple EM exposure and social isolation was significant. The odds of mental distress associated with multiple EM exposure were substantially higher when experienced in social isolation (OR = 7.23, [CI: 3.65, 14.3]) than in non-isolation (OR = 2.68, [CI: 1.66, 4.32]). Findings suggest the signified mental health risk of the co-experience of EM and social isolation and call attention to those who might silently suffer from EM in isolation. Comprehensive interventions that encompass both preventive measures and targeted support systems are in need.

